# Serum NT-Pro-BNP versus Noninvasive Bedside Inotropic Index in Paediatric Shock: A Contest of Myocardial Performance in Response to Fluid Loading

**DOI:** 10.1155/2021/7458186

**Published:** 2021-11-30

**Authors:** Antonius Hocky Pudjiadi, Tuty Rahayu, Stephanie Wijaya, Fatima Safira Alatas

**Affiliations:** ^1^Department of Child Health Cipto Mangunkusumo Hospital, Faculty of Medicine, Universitas Indonesia, Kota Depok, Indonesia; ^2^Pasar Rebo Regional General Hospital, Jakarta, Indonesia

## Abstract

**Background:**

Mild elevation of serum amino-terminal pro-B-type natriuretic peptide (NT-pro-BNP) is associated with myocardial dysfunction. A significantly lower Smith–Madigan inotropic index (SMII) has been shown to accurately represent cardiac contractility among heart failure subjects. We aim to monitor the effect of fluid resuscitation on cardiac function among paediatric patients by measuring serum NT-pro-BNP and SMII.

**Methods:**

This is an observational study on 70 paediatric shock patients. NT-pro-BNP and noninvasive bedside haemodynamic monitoring were done by using an ultrasonic cardiac output monitor (USCOM, USCOM, Sydney, Australia). The presence of cardiac diseases was excluded. SMII was obtained from the USCOM. An increase in the stroke volume index (SVI) of ≥15% indicates fluid responders. Measurements were taken before and after fluid loading.

**Results:**

Preloading NT-pro-BNP and SMII category were significantly different between the fluid responsiveness group, *p*=0.001 and *p*=0.004, respectively. Higher median NT-pro-BNP (preloading NT-pro-BNP of 1175.00 (254.50–9965.00) ng/mL vs. 196.00 (65.00–509.00) ng/mL, *p*=0.002) was associated with fluid nonresponders (subjects >12 months old). Preloading NT-pro-BNP <242.5 ng/mL was associated with fluid responders (AUC: 0.768 (0.615–0.921), *p*=0.003), 82.1% sensitivity, and 68.7% specificity for subjects >12 years old. Delta NT-pro-BNP in fluid responders (15.00 (−16.00–950.00) ng/mL) did not differ from fluid nonresponders (505.00 (−797.00–1600.00) ng/mL), *p*=0.456. Postloading SMII >1.25 W·m^−2^ was associated with fluid responders (AUC: 0.683 (0.553–0.813), *p* = 0.011), 61.9% sensitivity, and 66.7% specificity, but not preloading SMII. Fluid responders had a higher mean postloading SMII compared to nonresponders (1.36 ± 0.38 vs. 1.10 ± 0.34, *p*=0.006).

**Conclusion:**

Higher NT-pro-BNP and lower SMII in the absence of cardiac diseases were associated with poor response to fluid loading. The SMII is affected by low preload conditions.

## 1. Introduction

Nowadays, paediatric resuscitation guideline recommends the use of haemodynamic monitoring to guide fluid resuscitation [1]. Most haemodynamic measurements involve either invasive methods, such as arterial catheterization, central venous pressure (CVP), or echocardiography, which may only be available in the paediatric intensive care unit (PICU) [2]. A reliable, noninvasive, and rapid haemodynamic assessment of paediatric haemodynamic physiology is therefore needed to guide fluid resuscitation.

Serum level of amino-terminal pro-B-type natriuretic peptide (NT-pro-BNP) is found to increase in structural cardiac diseases, as well as cardiac failure [3, 4]. Its use has been previously validated for diagnosis and therapeutic guidance, as well as a prognostic value among heart failure subjects [5]. A grey area in NT-pro-BNP elevation has been shown to correlate with a mild degree of cardiac dysfunction, outside of clinical diagnosis of heart failure [6].

The ultrasonic cardiac output monitor (USCOM) is a noninvasive, bedside haemodynamic monitoring device that has been widely used to study haemodynamics in adults and paediatric patients [7, 8]. Its use in the paediatric population has been validated, and results have been compared to the pulmonary arterial catheter (PAC) thermodilution measurements [8]. Smith–Madigan inotropic index (SMII) can be derived from USCOM measurements and has been shown to be able to discriminate congestive heart failure subjects in adults as it reflects contractility [7].

The use of serum NT-pro-BNP and USCOM during fluid resuscitation has never been investigated. This study aims to assess the value of NT-pro-BNP and SMII in assessing myocardial response to fluid loading in paediatric shock patients.

## 2. Methods

We performed a prospective observational study at Pasar Rebo Regional Referral Hospital, a secondary general hospital in Jakarta, Indonesia, from March 2020 to March 2021. All data were recorded following written informed consent from parents or guardians. Ethical approval was obtained from the Ethics Committee of Faculty of Medicine, Universitas Indonesia (approval no. KET-553/UN.F1/ETIK/PPM.00.02/2019).

We enrolled paediatric patients older than 1 month old, indicated for fluid resuscitation due to the presence of hypotension and/or signs of impaired perfusion (tachycardia if mean heart rate >2SDs above normal for age; skin mottling or capillary refilling time >3 seconds) [9, 10]. Subjects were excluded if it was found that the patient had any contraindication to fluid loading (congenital heart disease, acute kidney injury, or chronic kidney disease), jugular vein distension, presence of gallop (third heart sound) or pulmonary rales on auscultation, abdominal ascites or hepatomegaly, or any other clinical impression of volume overload according to the attending physician, or any other available laboratory evidence of contraindication for fluid loading (X-ray, echocardiography, or ECG) [11]. Before fluid resuscitation, patients' vital signs were recorded, the haemodynamic assessment was performed by using the USCOM (USCOM^®^; USCOM Ltd., Sydney, Australia), and peripheral venous blood was collected for serum NT-pro-BNP and other laboratory examinations.

**Figure 1:**
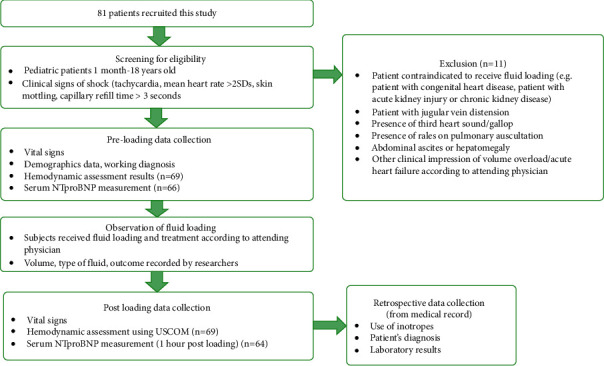
Flowchart of study design.

Researcher team who collected the sample and recorded the data did not make any clinical decision for subject's treatment. However, attending physicians were not blinded to the USCOM parameters measured and were able to use it for clinical purposes. Following fluid resuscitation (subjects no longer received fluid loading), vital signs and haemodynamic assessment were repeated by the respective attending clinician. One hour after fluid resuscitation ends, blood samples were collected for serum NT-pro-BNP measurements, during which subjects only received maintenance infusion. The flow of the study is presented in Figure 1.

Mean arterial pressure (MAP) was defined as (systolic blood pressure (BP) + 2 × diastolic BP)/3 and further categorized into percentiles [12]. Patients received treatment according to their clinical diagnosis. Patient demographic data, in-hospital mortality, use of inotropes, and types of shock were recorded retrospectively from patients' medical records. Since any structural heart anomalies affect the serum NT-pro-BNP level and SMII value, any subjects proven to have cardiac abnormalities during in-hospitalization were excluded.

### 2.1. Haemodynamic Monitoring

The haemodynamic assessment was performed using the USCOM (USCOM^®^; USCOM Ltd., Sydney, Australia) by trained professionals and measured three times at the aorta. We did not perform central venous pressure (CVP) measurement, and values were assumed to be 0. The inotropic index was expressed as the Smith–Madigan inotropic index (SMII) [7]. “Percentage of SMII” was defined as measured SMII-lower limit (according to the age category)/lower limit *×* 100%. SMII was further classified “low” percentage of SMII < −35% [7], otherwise as “normal.” “Delta SVI percentage” was defined as (postloading SVI − preloading SVI)/preloading SVI *×* 100%. Fluid responsiveness was defined as delta SVI percentage ≥+15%. Left ventricular end-diastolic volume index (LVEDVI) was calculated using inotropy 2009 software (USCOM^®^; USCOM Ltd., Sydney, Australia).

### 2.2. NT-Pro-BNP

Serum NT-pro-BNP was quantified using the electrochemiluminescence immunoassay (ECLIA) method and reagent pro-BNP II CalSet (Cat. no. 04842472190, Roche Diagnostics, Mannheim, Germany) on an Elecsys Modular E170 platform (Roche Diagnostics, Mannheim, Germany), performed at a local commercial laboratory partner, Prodia Clinical Laboratory (Prodia Utama Ltd., Jakarta, Indonesia). Serum NT-pro-BNP values were categorized as “high” if they exceed the upper limit of the normal range according to the age group [13]. “Delta NT-pro-BNP” was defined as postloading NT-pro-BNP − preloading NT-pro-BNP while “delta NT-pro-BNP” as delta NT-pro-BNP/preloading NT-pro-BNP × 100%. In calculating delta NT-pro-BNP, subjects with preloading NT-pro-BNP of >70000 were excluded as the limit of detection of NT-pro-BNP was 50–70000 pg/mL.

### 2.3. Statistical Analysis

Data were computed into Excel and analysed using SPSS IBM version 24.0 (IBM Corp, Armonk, USA). Variable frequency was expressed as frequency and percentage. Shapiro–Wilk test was used to assess data distribution, in which nonparametric data were expressed as median (interquartile range, IQR). Statistical significance was set at 5%. Chi-square, Fisher's exact test, and independent Kruskal–Wallis test were used to analyse categorical data association. Between-group comparison of numerical data was conducted using Mann–Whitney U or unpaired *T*-test. Bivariate correlation analysis was performed using Spearman's correlation, while the multivariate linear model was used for multivariate analysis. The area under the curve (AUC) was obtained by constructing a receiver operating characteristic (ROC) curve, and optimum cutoff was identified using the intersection point by plotting sensitivity against specificity.

## 3. Results

### 3.1. Subject Characteristics

Overall, 61.76% of subjects had their SVI increased by ≥15% upon fluid loading. Subject characteristics are demonstrated in Table 1. Fluid responsiveness did not differ across age, gender, nutritional status, and amount of fluid administered. Inotrope was eventually administered in 47.06% of subjects. Types of shock were significantly associated with fluid responsiveness (*p*=0.026); among all subjects, 15 (22.06%) were diagnosed as septic shock, 29 (42.65%) were diagnosed as dengue shock, 24 (35.29%) were diagnosed as other types of shock (hypovolemia due to gastrointestinal loss and haemorrhagic), and none were diagnosed as anaphylactic or neurologic shock. Normal preloading serum NT-pro-BNP was associated with fluid responsiveness (*p*=0.001), while a low preloading SMII was associated with fluid responsiveness (*p*=0.004) (Table 1).

### 3.2. Serum NT-Pro-BNP

**Figure 2:**
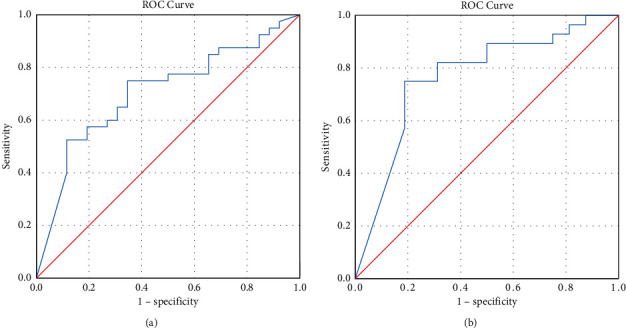
Preloading serum NT-pro-BNP in predicting fluid responsiveness. (a) ROC curve for all subjects (*n* = 66). (b) ROC curve for subjects >12 months old (*n* = 44).

Using all subjects' numerical value of preloading serum NT-pro-BNP, we obtained an optimum cutoff of NT-pro-BNP <725.5 ng/mL to discriminate fluid responders with an AUC of 0.705 (95% CI: 0.578–0.833, *p*=0.005), a specificity of 75%, and a sensitivity of 65.4%. Excluding subjects under 12 months old, we obtain an optimum cutoff of NT-pro-BNP of <242.5 ng/mL to discriminate fluid responders with an AUC of 0.768 (95% CI: 0.615–0.921, *p*=0.003), a sensitivity of 82.1%, and a specificity of 68.7% (Figure 2).

Under numerical analysis, fluid responsiveness was associated with lower median serum NT-pro-BNP before and after loading only for subjects >12 years old (Table 2). Both delta NT-pro-BNP and delta NT-pro-BNP percentage were lower in fluid responders (>12 months old); however, neither were statistically significant.

### 3.3. Smith–Madigan Inotropic Index

**Figure 3:**
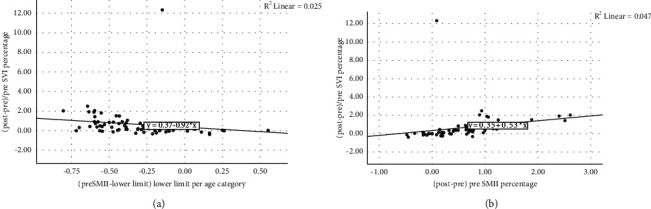
Linear correlation between SMII and SVI. (a) Percentage of preloading SMII against delta SVI percentage. (b) Percentage of delta SMII against percentage of delta SVI.

A low preloading SMII was associated with fluid responsiveness (*p*=0.004), with a significant negative correlation between percentage preloading SMII from the lower limit and percentage of delta SVI ((postloading − preloading SVI)/preloading SVI × 100%) (Spearman's correlation coefficient: −0.546, *p* ≤ 0.001). On the contrary, despite the nonsignificant correlation, percentage of postloading SMII from the lower limit had a positive correlation coefficient with a percentage of delta SVI (Spearman's correlation coefficient: 0.147, *p*=0.236). We observed that delta SMII percentage had a moderate positive correlation with delta SVI percentage (Spearman's correlation coefficient: 0.726, *p* ≤ 0.001) (Figure 3).

**Figure 4:**
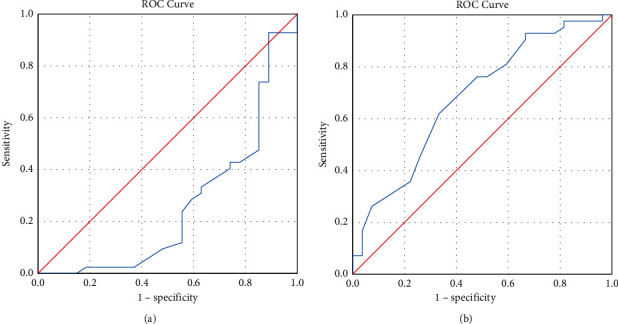
SMII in predicting fluid responsiveness. (a) ROC of preloading SMII in predicting fluid responsiveness (*n* = 68). (b) ROC of postloading SMII in predicting fluid responsiveness (*n* = 68).

Preloading SMII poorly discriminates fluid responders in our subjects, with an AUC of 0.261 (95%CI: 0.132–0.390, *p*=0.001). However, postloading SMII was able to discriminate fluid responders (AUC: 0.683, 95% CI: 0.553–0.813, *p*=0.011) with an optimum cutoff of >1.25, sensitivity of 61.9%, and specificity of 66.7% (Figure 4).

Since NT-pro-BNP values vary greatly, we performed log_*e*_ (ln) transformation of preloading NT-pro-BNP and used ln(242.5) = 5.49 as a *y*-axis reference line. There was no significant correlation between preloading NT-pro-BNP and postloading SMII in subjects aged >12 months old. Under categorical analysis, the postloading SMII category was not associated with the preloading NT-pro-BNP classification (*p*=0.596).

### 3.4. Pre- and Postloading Haemodynamic Parameters

Fluid responders were associated with a lower median preloading CI (*p* ≤ 0.001), median preloading SVI (*p*=0.001), and lower mean preloading SMII (*p* ≤ 0.001). However, using postloading values, only higher SMII was associated with fluid responders (*p*=0.006) (Table 3).

### 3.5. Multiple Linear Regression

Since preloading SMII had a significant correlation with preloading SVI, we included the percentage of preloading SVI into the multilinear regression. None of the variables were significantly associated with delta SVI percentage, apart from preloading SVI (percentage from the lower limit) (Table 4). Upon collinearity analysis, preloading SMII (percentage from the lower limit) had a positive linear correlation with preloading SVI (percentage from the lower limit), with a correlation coefficient of 0.671.

## 4. Discussion

NT-pro-BNP has been widely used to diagnose congestive heart failure in children [3]. SMII has also been used to describe CHF subjects in adult studies [4]. This study was intended to monitor cardiac performance before and after fluid resuscitation using both biomarker (NT-pro-BNP) and bedside haemodynamic marker (SMII) in non-CHF subjects. Overall, we found consistently higher NT-pro-BNP among fluid nonresponders and a low SMII (after loading) to be associated with fluid nonresponders.

Similar to previous findings, we also found a negative correlation between NT-pro-BNP and age [13]. This can be explained by increased adiposity and renal clearance of NT-pro-BNP as subjects' age increases [14]. NT-pro-BNP had no association with fluid responsiveness among infant subjects, possibly due to the wide normal range of NT-pro-BNP for this age group and the limited sample size.

An interesting observation in this study was that the lower NT-pro-BNP value was consistently associated with fluid responders among subjects >12 months old. NT-pro-BNP is associated with not only HF of cardiac structural origin but also other conditions such as sepsis and fever [15, 16]. Further studies in adult patients undergoing hemodialysis consistently use NT-pro-BNP as a marker of intravascular volume [17]. Elevated NT-pro-BNP can be caused by increased production, decreased clearance, or both and could be affected by the use of beta-blockers, cardiac glycosides, cardiotoxic substances, and those affecting renal clearance [15, 16]. Among our subjects, NT-pro-BNP may be higher in patients nonresponsive to fluid loading as those patients may have received fluid bolus prior to admission (especially true for referral patients), hence higher NT-pro-BNP detected, or in patients in which cardiac dysfunction was the major cause of haemodynamic failure, such as sepsis with impaired systolic/diastolic function. Regardless of the cause of shock, we found that a cutoff of NT-pro-BNP <242.5 pg/mL was able to discriminate positive responders upon fluid resuscitation. This is much lower than previous studies [5, 16, 18]. However, since we calculated the sample size for categorical analysis, further study with much larger sample size is needed to obtain a more reliable cutoff point to predict a positive response to fluid resuscitation, preferably for each age group category.

Another novel observation in this study was the value of postloading SMII as an inotropic index in discriminating fluid responders among shock subjects. Preloading SMII could not be used as we found that SMII is preload dependent under hypovolemic conditions. This was proven as lower preloading SMII percentage had a strong positive correlation with preloading SVI percentage, and delta SMII percentage had a strong positive correlation with delta SVI percentage. We think that this is due to contractility measured by the SMII which was derived from the SVI measured at the aortic notch via the Doppler ultrasound velocity-time integral. Postloading SMII with a cutoff of >1.25 was able to discriminate fluid responders. This cutoff is lower than the normal SMII range for age 3 months old and older according to the manufacturer's reference range. Furthermore, we showed that postloading SMII was significantly different between fluid responders and nonresponders compared to other markers such as postloading CI (Table 3). This implies that, aside from hypovolemia, SMII value can be used to estimate the inotropic index.

Despite both preloading NT-pro-BNP and postloading SMII being able to discriminate fluid responders, we found that preloading NT-pro-BNP was not associated with postloading SMII. On the Frank–Starling curve, both preload and inotropy affect the curve independently [19]. Based on our subjects, we found that NT-pro-BNP has higher sensitivity compared to SMII in identifying fluid responders. It is possible that NT-pro-BNP release occurs earlier before the decrease in the inotropic index occurs. A previous study on the grey zone of NT-pro-BNP advised not to regard it as a negative result. Its concentration relationship with HF severity might imply milder severity of cardiac involvement and warrants further investigation [6]. Furthermore, the cutoff of NT-pro-BNP obtained in our study is lower than that to diagnose congestive HF, while the cutoff of the SMII is significantly lower than the lower limit for all subjects, representing higher severity of contractility dysfunction. However, further study is needed to clarify this relationship.

Upon multiple linear regression, we identified that the preloading SVI percentage (percentage of the lower limit − preloading SVI) was the only significant factor in determining the positive response to fluid resuscitation. This is especially true as a lower preloading SVI would yield a higher percentage of SVI increase upon loading. We also found a moderate positive linear correlation between preloading SVI and preloading SMII even after adjusting for other variables. This further supports the finding of SMII calculation to be affected by low preload status.

In summary, this study found that NT-pro-BNP was able to identify fluid responders among paediatric shock patients. However, since it is a serum biomarker, its use in aiding clinical decisions is limited. Using bedside haemodynamic monitoring, we also found that the inotropic index, SMII, could not be used to predict a positive response to fluid resuscitation since it is affected by the SVI. SMII was not useful in identifying potential fluid responders. However, we found that the SMII can potentially be used for further study revolving around inotropy (cardiac contractility) as it may be a good representative of the inotropic index exclusive of hypovolemia. There were also some limitations to this study. Firstly, the small sample size was only able to determine the association between categorical variables. A bigger sample size is needed to obtain a more accurate cutoff for NT-pro-BNP and SMII across age categories. Secondly, USCOM was performed by a team of trained paediatricians; however, interoperator variability was still unavoidable. Thirdly, we did not adjust for the subjects' acute clinical conditions that might have affected NT-pro-BNP values. A previous study demonstrated that acute, noncardiac diseases and sepsis were linked to increased NT-pro-BNP, albeit much lower than the cutoff for HF; this might affect the cutoff obtained in the study due to heterogeneity of shock [15, 16]. Lastly, we observed a trend of higher increment in NT-pro-BNP upon loading among fluid nonresponders; however, this association remains underexplored due to our small sample size.

Our study was the first to demonstrate the use of NT-pro-BNP and SMII in monitoring response to fluid resuscitation in the paediatric population in the absence of cardiac diseases. We found that elevated NT-pro-BNP was associated with a poor response to fluid loading. We also show that a higher SMII value, as a measure of cardiac inotropy, was associated with a desirable outcome to fluid resuscitation; however, its value during low preload condition may be falsely low.

## Figures and Tables

**Table 1:** Subject characteristics.

Subject characteristics	Fluid nonresponders (*n* = 26)		Fluid responders (*n* = 42)		*p* value
	*n*	%	*n*	%	
Age in months (median, IQR)	27.00 (8.75–75.50)		51.00 (12.00–126.00)		0.180
Gender (male)	14	53.84	20	47.62	0.334
*Shock category*					
Septic shock	9	34.62	6	14.29	0.026^*∗*^
Dengue shock	6	23.08	23	54.76	
Others (hypovolemia and hemorrhagic)	11	42.31	13	30.95	

*Nutritional status (BW/ideal BW)*					
Malnutrition (*z* < −3SD)	4	15.38	6	14.29	0.383^c^
Undernourished (−3SD < *z* < −2SD)	5	19.23	7	16.67	
Normal (−2SD < *z* < +2SD)	11	42.31	21	50.00	
Overweight (+2SD < *z* < +3SD)	2	7.69	0	0.00	
Obese (*z* > +3SD)	4	15.38	8	19.05	

*Total fluid loading*					
<20 ml/kg BW	6	23.08	11	26.19	0.972^c^
20–<40 ml/kg BW	13	50.00	20	47.62	
40–<60 ml/kg BW	5	19.23	8	19.05	
>60 ml/kg BW	2	7.69	3	7.14	

Inotropic use, yes	10	38.46	22	52.38	0.534
Preloading MAP percentile (median, IQR)	30.00 (7.50–65.00)		50.00 (20.00–72.50)		0.567
Preloading NT-pro-BNP, normal^b^	7	26.92	29	69.05	0.001^*∗*^
Preloading SMII, normal	19	73.08	14	33.33	0.004^*∗*^
Preloading LVEDVI^b^ (median, IQR)	70.75 (57.05–78.25)		67.20 (56.78–76.20)		0.757

BW: body weight; MAP: mean arterial pressure; SMII: Smith–Madigan inotropic index; LVEDVI: left ventricular end-diastolic volume. Categorical data were analysed using chi-square unless stated otherwise, numerical data were analysed using independent Kruskal–Wallis test, and ^*∗*^*P* value <0.05; ^a^preloading MAP percentage from 64 subjects; ^b^preloading NT-pro-BNP percentage from 64 subjects; ^c^Fisher's exact test; ^d^independent sample *T*-test.

**Table 2:** Comparison of serum NT-pro-BNP according to fluid responsiveness.

	Fluid nonresponders	Fluid responders	*p* value
*Preloading NT-pro-BNP, ng/mL*			
>1–12 months old (*n* = 22)	4352.00 (2607.00–6469.00)	1329.00 (550.00–5126.00)	0.145
>12 months old (*n* = 44)	1175.00 (254.50–9965.00)	196.00 (65.00–509.00)	0.002^*∗*^

*Postloading NT-pro-BNP, ng/mL*			
>1–12 months old (*n* = 23)	2050.00 (1806.00–6323.00)	3970.00 (649.00–7215.00)	0.565
>12 months old (*n* = 41)	1680.00 (472.00–7603.00)	151.00 (74.00–1790.00)	0.004^*∗*^

*Delta NT-pro-BNP, ng/mL*			
>1–12 months old (*n* = 19)	−801.00 (−3523.00–1897.00)	719.00 (−287.00–6471.00)	0.561
>12 months old (*n* = 20)	505.00 (−797.00–1600.00)	15.00 (−16.00–950.00)	0.456

*Delta NT-pro-BNP percentage*			
>1–12 months old (*n* = 19)	−0.31 (−0.78–1.94)	0.18 (−0.40–1.56)	0.561
>12 months old (*n* = 38)	0.39 (−0.41–3.63)	0.15 (−0.14–1.28)	0.722

Nonparametric data were presented as median (IQR), ^*∗*^Mann–Whitney U, and *P* value <0.05.

**Table 3:** Comparison between preloading and postloading haemodynamic parameters based on fluid responsiveness.

	Fluid nonresponders	Fluid responders	*p* value
*Preloading haemodynamic parameters*			
Preloading CI	3.95 (280–4.70)	2.50 (1.50–3.33)	≤0.001^*∗*^
Preloading SVI, mL/m^2^	28 .00 (21.25–31.00)	16.00 (14.00–22.50)	0.001^*∗*^
Preloading SVRI, ds cm^−5^	1444.00 (1180.75–2265.50)	2841.50 (1700.75–3890.50)	0.083
Preloading SVV^a^, %	49.77 ± 24.64	51.33 ± 19.39	0.125
Preloading SMII^a^, W/m^2^	1.08 ± 0.41	0.75 ± 0.21	≤0.001^*∗*^
Preloading DO_2_, mL/min	286.00 (195.25–481.25)	240.00 (176.50–455.00)	0.199

*Postloading haemodynamic parameters*			
Postloading CI	3.70 (2.98–4.40)	3.70 (3.00–4.83)	0.690
Postloading SVI, mL/m^2^	24.00 (18.75–30.00)	30.00 (23.75–35.00)	0.123
Postloading SVRI, ds cm^−5^	1468.00 (1130.50–1918.50)	1738.00 (1204.25–2389.50)	0.428
Postloading SVV, %	35.55 (27.50–49.25)	32.00 (22.25–49.25)	0.390
Postloading SMII^a^, W/m^2^	1.10 ± 0.34	1.36 ± 0.38	0.006^*∗*^
Postloading DO_2_, mL/min	284.00 (155.50–471.00)	489.00 (251.25–758.50)	0.083

SVI: stroke volume index; SVRI: systemic vascular resistance index; SVV: stroke volume variation; SMII: Smith–Madigan inotropic index; DO_2_: oxygen delivery.

**Table 4:** Multilinear correlation between variables against delta SVI.

	Unstandardized coefficients		Standardized coefficients		Sig.
	*B*	Std. error	Beta	*t*	
Constant	−9.909	6.285		−1.577	0.120
MAP percentile	0.003	0.041	0.008	0.066	0.948
Preloading LVEDVI	0.136	0.072	0.231	1.889	0.064
Preloading SMII (%)^a^	1.444	3.864	0.060	0.374	0.710
Preloading NT-pro-BNP	−3.333*E *− 6	0.000	−0.005	−0.041	0.968
Preloading SVI (%)^b^	−16.182	5.448	−0.515	−2.970	0.004^*∗*^

MAP: mean arterial pressure; LVEDVI: left ventricular end-diastolic volume index; SMII: Smith–Madigan inotropic index; SVI: stroke volume index. ^a^(preloading SMII-lower limit)/lower limit according to age *×* 100%. ^b^(preloading SVI-lower limit)/lower limit according to age *×* 100%.

## Data Availability

The data used to support the findings of this study are available from the corresponding author upon reasonable request.
